# Anti-COVID property of subcutaneous ivermectin in synergy with zinc among midlife moderately symptomatic patients: a structured summary of a study protocol for a randomised controlled trial

**DOI:** 10.1186/s13063-021-05487-z

**Published:** 2021-09-06

**Authors:** Shoaib Ashraf, Sohaib Ashraf, Iqra Farooq, Sidra Ashraf, Moneeb Ashraf, Muhammad Ahmad Imran, Larab Kalsoom, Rutaba Akmal, Muhammad Ghufran, Sundas Rafique, Muhammad Kiwan Akram, Zaigham Habib, Uzma Nasim Siddiqui, Ammara Ahmad, Shahroze Arshad, Muhammad Abdul Rehman Virk, Mehak Gul, Abeer bin Awais, Muhammad Hassan, Syed Sami Hussain Sherazi, Zartasha Safdar, Isra Munir, Hamna Khalid, Khalid Munir, Nighat Majeed, Yaser Masuod Alahmadi, Ayesha Humayun, Qazi Abdul Saboor, Ali Ahmad, Muhammad Ashraf, Mateen Izhar, DOCTORS LOUNGE Consortium, Abubakr Hilal, Arz Muhammad, Zeeshan Shaukat, Sohail Ahmad, Kanwal Hayat, Ghazala Amjad, Misbah Kousar, Hadiqa Tul Hafsa, Zawar Ahmad Choudhary, Umair Hafeez, Tayyab Mughal, Noman Khalid, Qurat-ul-Ain Iqbal, Roa Umer, Tayyaba Muzafar, Sibgha Zulfiqar, Saadia Shahzad Alam, Saulat Sarfraz, Muhammad Imran Anwar, Amber Malik, Talha Mahmud, Adeen Akmal, Muhammad Faisal Nadeem, Nazish Matti, Muhammad Azam, Nighat Majeed, Ali Arshad, Khawar Nawaz, Muhammad Ismail Khalid Yousaf, Aadil Maqsood Atif Amin Baig, Muhammad Bilal, Muhammad Nauman Zahid

**Affiliations:** 1 Wellman Center for Photomedicine, Massachusetts General Hospital, Harvard Medical School, Boston, USA; 2 Department of Pathobiology, Riphah International, Lahore, Pakistan; 3 Department of Cardiology, Shaikh Zayed Post-Graduate Medical Institute, Lahore, Pakistan; 4 Department of Paediatric Surgery, Children Hospital, Lahore, Pakistan; 5 Institute of Biochemistry and Biotechnology, University of Veterinary and Animal Sciences, Lahore, Pakistan; 6 Department of Pharmacology, Mayo Hospital, Kingedward Medical University, Lahore, Pakistan; 7 Department of Microbiology, Shaikh Zayed Post-Graduate Medical Institute, Lahore, Pakistan; 8 Department of Medicine, Services Institute of Medical Sciences, Lahore, Pakistan; 9 Department of Medicine, Sahara Medical College, Narowal, Pakistan; 10 Department of West Medicine, Mayo Hospital, King Edward Medical University, Lahore, Pakistan; 11 Department of animal nutrition, University of veterinary and animal sciences, Lahore, Pakistan; 12 Department of Orthopedics, Shaikh Zayed Post-Graduate Medical Institute, Lahore, Pakistan; 13 Department of Medicine, Shaikh Zayed Post-Graduate Medical Institute, Lahore, Pakistan; 14 Department of Radiology, Shaikh Zayed Post-Graduate Medical Institute, Lahore, Pakistan; 15 Department of Pharmacology and Toxicology, University of veterinary and animal sciences, Lahore, Pakistan; 16 Department of Restorative & Preventative Sciences University of Pennsylvania School of Dental Medicine University of Pennsylvania, Philadelphia, PA USA; 17 Specialized Sciences Post-Baccalaureate Program, College of Liberal Arts & Sciences, University of Pennsylvania, Philadelphia, PA USA; 18 Department of Internal Medicine, Services Institute of Medical Sciences, Lahore, Pakistan; 19 Clinical and Hospital Pharmacy Department, College of Pharmacy, Taibah University Madinah, Medina, Kingdom of Saudi Arabia; 20 Department of Community Medicine and Public Health, Shaikh Zayed Post-Graduate Medical Complex, Lahore, Pakistan; 21 Department of Microbiology, Infectiology and Immunology, Centre Hospitalier Universitaire (CHU) Sainte Justin/University of Montreal, Montreal, Canada

**Keywords:** Ivermectin, Zinc, Subcutaneous Ivermectin, Pakistan, COVID-19, Randomised controlled trial, protocol

## Abstract

**Objectives:**

The study objective is to quantify the effectiveness of ivermectin (subcutaneous/oral IVM) in the presence or absence of zinc (Zn) for clinical and radiological improvement in coronavirus disease 2019 (COVID-19) patients with moderate severity.

**Trial design:**

This quadruple-blinded, placebo-controlled randomized clinical trial will be a multiarmed multi-centered study with superiority framework.

**Participants:**

Quinquagenarian and sexagenarian patients with moderate COVID-19 symptoms and positive severe respiratory syndrome coronavirus -2 (SARS-CoV-2) PCR will be included. Participants with co-morbidities and pregnant women will be excluded.

Patient recruitment will be done in Shaikh Zayed Medical Complex, Doctors Lounge and Ali Clinic in Lahore (Pakistan).

**Intervention and comparator:**

The registered patients will be allocated in 6 groups (30 participants each). Patients will be taking subcutaneous IVM at 200 μg/kg/48 h (Arm A) or subcutaneous IVM at 200 μg/kg/48 h and oral Zn 20mg/8 h (Arm B) or oral IVM at 0.2 mg/kg/day (Arm C) or oral IVM at 0.2 mg/kg/day and oral Zn 20mg/8 h (Arm D) or alone oral Zn 20mg/8 h (Arm E) or placebo alone (Arm X). Patients in all arms will receive standard care and respective placebo (empty capsule 8 hourly and/or subcutaneous normal saline 2ml/48 h).

**Main outcomes:**

Primary endpoints will be duration of symptomatic phase and SARS-CoV-2 clearance along with high resolution CT (HRCT) chest score and clinical grade scale (CGS) on day 6. 30-day mortality will be documented as a secondary endpoint. SARS-CoV-2 clearance will be calculated by second PCR on day 7. HRCT chest score will be measured by the percentage and lung lobes involvement on day 6 with a maximum score of 25. CGS will be recorded on a seven-point scale; grade 1 (not hospitalized, no evidence of infection and resumption of normal activities), grade 2 (not hospitalized, but unable to resume normal activities), grade 3 (hospitalized, not requiring supplemental oxygen), grade 4 (hospitalized, requiring supplemental oxygen), grade 5 (hospitalized, requiring nasal high-flow oxygen therapy and/or noninvasive mechanical ventilation), grade 6 (hospitalized, requiring ECMO and/or invasive mechanical ventilation) and grade 7 (death).

**Randomisation:**

A simple lottery method will be used to randomly allocate scrutinized patients in 1:1:1:1:1:1 ratio in 6 groups.

**Blinding (masking):**

Patients, primary care physicians, outcome assessors and the data collection team will be blinded.

**Numbers to be randomised (sample size):**

180 participants will be randomized into six arms with five investigational and one placebo group.

**Trial Status:**

Institutional Review Board Shaikh Zayed Post-Graduate Medical Complex, Lahore, Pakistan has approved the protocol (version 2.3) with ID SZMC/IRB/Internal0056/2020. The trial was approved on July 14, 2020, and enrolment started on July 30, 2020. The estimated completion date is October 30, 2021.

**Trial registration:**

Clinical Trial has been retrospectively registered on www.clinicaltrials.gov with registration ID NCT04472585 dated July 16, 2020.

**Full protocol:**

The full protocol is attached as an additional file, accessible from the Trials website (Additional file 1). With the intention of expediting dissemination of this trial, the conventional formatting has been eliminated; this Letter serves as a summary of the key elements of the full protocol. The study protocol has been reported in accordance with the Standard Protocol Items: Recommendations for Clinical Interventional Trials (SPIRIT) guidelines.

**Supplementary Information:**

The online version contains supplementary material available at 10.1186/s13063-021-05487-z.

## Supplementary Information

**Additional file 1.** Full protocol.

## Data Availability

Dr. Sohaib Ashraf will have access to the final trial dataset, and this could be available from the author on reasonable request, but the dataset is subject to data protection regulations. (Email address: sohaib@skzmdc.edu.pk Mobile Number: +1 (857) 316 7995)

