# Supplementary material for: Anti-COVID property of subcutaneous ivermectin in synergy with zinc among midlife moderately symptomatic patients: a structured summary of a study protocol for a randomised controlled trial
Source: Trials. 2021 Sep 6;22:591. doi: 10.1186/s13063-021-05487-z (PMC8419386; doi:10.1186/s13063-021-05487-z)
Supplement: Supplementary file 1 — Additional file 1. Full protocol. [file 13063_2021_5487_MOESM1_ESM.pdf]

Title

Efficacy of Subcutaneous Ivermectin with or without Zinc in Mild to Moderate COVID-19 patients

Trial Name

SIZI-COVID-PK

Study Chair:

Prof. Dr. Muhammad Ashraf, PhD (University of Minnesota)
Designation: Professor Emeritus Department of Pharmacology & Toxicology
Institutional Affiliation: University of Veterinary & Animal Sciences, Lahore, Pakistan

Study Chair:

Dr. Shoaib Ashraf, PhD (McGill University)
Designation: Research Associate Parasitology & Pharmacology (PhD thesis on Ivermectin)
Institutional Affiliation: Massachusetts Hospital Harvard University, Boston, USA

Study Principal Investigators:

1. **Dr. Moneeb Ashraf, M.Phil. (Federal Post-Graduate Medical Institute)**
   Designation: Associate Professor Pharmacology
   Institutional Affiliation: Mayo Hospital, Kingedward Medical University, Lahore, Pakistan
2. **Dr. Sohaib Ashraf, MBBS (Shaikh Khalifa Bin Zayed Al-Nahyan Medical & Dental College)**
   Designation: MD Post-Graduate Cardiology trainee
   Institutional Affiliation: Shaikh Zayed Medical Complex, Lahore, Pakistan

Study Co-Investigators:

1. **Prof. Dr. Ali Ahmad, PhD (Virology)**
   Designation: Professor of Immunology & Virology
   Institutional Affiliation: University of Montreal, Sainte Justin Hospital, Canada.
2. **Dr. Talha Mahmood, MD (Pulmonology)**
   Designation: Head of Department of Pulmonology
   Institutional Affiliation: Shaikh Zayed Medical Complex, Lahore, Pakistan
3. **Dr. Qazi Abdul Saboor, FCPS(Cardiology)**
   Designation: Associate Professor Cardiology
   Institutional Affiliation: Shaikh Zayed Medical Complex, Lahore, Pakistan.
4. **Dr. Uzma Mamoon, FCPS(Medicine)**
   Designation: Assistant Professor Medicine
   Institutional Affiliation: Shaikh Zayed Medical Complex, Lahore, Pakistan

Site Investigators:

1. **Dr. Zain-ul-Abdin, MRCP**
   Institutional Affiliation: Shaikh Zayed Hospital, Lahore, Pakistan.
2. **Dr. Ayesha Khaqan, MRCP**
   Institutional Affiliation: Shaikh Zayed Hospital, Lahore, Pakistan.
3. **Dr. Muhammad Ahmad Imran, MBBS**
   Institutional Affiliation: Shaikh Zayed Medical Complex, Lahore, Pakistan
4. **Dr Larab Kalsoom, MBBS**
   Institutional Affiliation: Services Institute of Medical Sciences, Lahore, Pakistan
5. **Ghazala Amjad, BSc (Nursing)**
   Institutional Affiliation: Shaikh Zayed Hospital, Lahore, Pakistan
6. **Kanwal Hayat, BSc (Nursing)**
   Institutional Affiliation: Shaikh Zayed Hospital, Lahore, Pakistan

INTERNATIONAL COLLABORATORS:

1. **Dr. Umer Naveed, PhD**
   Designation: Research Scholar in Parasitology & Pharmacology (numerous research publications on Ivermectin (sub-cutaneous)
   Institutional Affiliation: Royal (Dick) School of Veterinary Studies and Roslin Institute, Scotland, UK.
2. **Dr. Fazli Mabood, PhD**
   Designation: Research Scientist Microbiology
   Institutional Affiliation: CHU Sainte-Justine Hospital, University of Montreal, Canada.
3. **Dr. Muhammad Bilal, M.Phil.**
   Designation: Research Associate Virologist
   Institutional Affiliation: McGill University, Sainte-Anne-de-Bellevue, Quebec, Canada.

LEAD BIOSTATITICIAN:

Prof. Dr. Muhammad Azam, PhD
Designation: Dean Faculty of Life Sciences Business Management, Professor of biostatistics, Lead Biostatistician
Institutional Affiliation: University of Veterinary & Animal Sciences, Pakistan

RESEARCH PHARMACISTS & DRUG SAFETY PANELISTS:

1. **Dr. Asad-ullah, Pharm.D**
   Designation: Assistant director Drug Regulation Authority Pakistan
   Institutional Affiliation: Drug Regulation Aurthority Pakistan (DRAP)
2. **Dr Zaka-ur-Rehman, PhD**
   Designation: Chief operating officer, Pakistan drug testing and research center
   Institutional Affiliation: Sundar Industrial Estate, Lahore, Pakistan.
3. **Dr. Syed Sami Hussain Sherazi**, **PharmD**
   Designation: Department of Pharmacology and Toxicology
   Institutional Affiliation: University of veterinary and Animal Sciences
4. **Dr. Zartasha Safdar, PharmD**
   Designation: Department of Pharmacology and Toxicology
   Institutional Affiliation: University of veterinary and Animal Sciences

CLINICAL EXPERT PANEL:

- **Prof. Dr. Amber Malik, MRCP (Cardiology)**
  Designation: Professor, Head of department Cardiology
  Institutional Affiliation: Shaikh Zayed Medical Complex, Lahore, Pakistan
- **Dr. Nighat Masood, FCPS (Medicine)**
  Designation: Associate Professor of Medicine unit 2
  Institutional Affiliation: Services Institute of Medical Sciences, Lahore, Pakistan.

TRIAL STEERING COMMITTEE

- **Prof. Dr. Roger Prichard, PhD**
  Designation: James McGill Professor of Parasitology & Pharmacology
  Institutional Affiliation: Institute of Parasitology, McGill University, Sainte-Anne-de-Bellevue, Quebec, Canada
- **Prof. Dr. Xin Zhao, PhD.**
  Designation: James McGill Professor
  Institutional Affiliation: McGill University, Sainte-Anne-de-Bellevue, Quebec, Canada
- **Prof. Dr. Mateen Izhar, PhD.**
  Designation: Professor of Microbiology, Chairman & Dean Shaikh Zayed Hospital.
  Institutional Affiliation: Shaikh Zayed Medical Complex, Lahore, Pakistan.
- **Prof. Dr. Muhammad Suhail, M.Phil.**
  Designation: Chairman Institutional Review Board & Head of Department of Anatomy
  Institutional Affiliation: Shaikh Zayed Medical Complex, Lahore, Pakistan
- **Prof. Dr. Amber Malik, MRCP**
  Designation: Professor, Head of department Cardiology
  Institutional Affiliation: Shaikh Zayed Medical Complex, Lahore, Pakistan
- **Prof. Dr. Muhammad Azam, PhD.**
  Designation: Dean Faculty of Life Sciences Business Management, Professor of biostatistics, Lead Biostatistician
  Institutional Affiliation: University of Veterinary & Animal Sciences, Pakistan
- **Prof. Dr. Ali Ahmad, PhD (Virology)**
  Designation: Professor of Immunology & Virology
  Institutional Affiliation: University of Montreal, Sainte Justin Hospital, Canada.
- **Prof. Dr. Muhammad Ashraf, PhD**
  Designation: Professor Emeritus of Pharmacology & Toxicology
  Institutional Affiliation: University of Veterinary & Animal Sciences, Pakistan

Abstract

Background: Since the first report from Wuhan China in December 2019, Corona Virus Disease-19 (COVID-19) caused by the novel coronavirus, SARS-CoV-2, has spread globally infecting millions. An effective response requires development of a successful treatment regimen on urgent basis while maintaining the clinical safety of patients. Despite a wide range of advised options for the treatment of COVID-19, a single strategy to tackle this pandemic remains elusive, thus far. That's why we are conducting a clinical trial to find out the efficacy of Ivermectin (sub-cutaneous/oral) with or without zinc in treating the COVID-19 patients.

Objective: The objective of this study is to measure the effect of Ivermectin with or without zinc and Iodine complex in treating the COVID-19 patients to clear viral load of SARS-CoV-2 along with reduction in severity of symptoms and length of hospitalization of patients with COVID-19.

Method: The proposed study is a placebo-controlled, add-on, randomized trial using parallel group designs. This is a close-label and adaptive design with superiority framework. It will be conducted in Shaikh Zayed Hospital. The study will have 5 interventional arms (A-E) and 1 control (placebo) arm containing 30 patients in each group. Arm A will be administered IVM subcutaneously at 200 µg/kg/48 h, Arm B will receive both IVM subcutaneously at 200 µg/kg/48 h and Zn orally 20 mg/every 8 h, Arm C will be given IVM orally at 200 µg/kg/day, Arm D will be given both IVM (orally at 200 µg/kg/day) and Zn (orally 20 mg/every 8 h) and Arm E will only receive Zn (orally 20 mg/every 8 h). Arm X will be standard care with placebo (subcutaneous normal saline 2 ml 48 h and empty capsule every 8 h). Data will be collected on self-constructed, close-ended questionnaires after obtaining written consent. Data will be analyzed using SAS. COVID-19 patients will be monitored by qRT-PCR and HRCT (High Resolution Computed Tomography) chest. In addition to these clinical improvements, duration of hospital stay, and mortality benefits will be analyzed in both groups.

Discussion: The study is designed to measure the superior efficacy of Ivermectin (oral/sub-cutaneous) with and without zinc in treating the COVID-19 positive patients with mild to moderate symptoms. This combination is hypothesized to improve different parameters like rapid viral load reduction and a negative qRT-PCR, rapid clinical progress, clear HRCT chests, low mortality rates and reduction in hospitalization. The trial will aid in devising a better strategy to cope COVID-19 in a relatively inexpensive and accessible. The implications are global, and this could prove itself to be the most manageable intervention against COVID-19 especially for patients from limited-resource countries with deprived socioeconomic statuses.

Registration Number: Clinical Trial registration will be done at www.clinicaltrials.gov

Keywords

Ivermectin, Zinc, Clinical Trial, COVID-19, SARS-CoV-2

Administrative information

Title(1)	Randomized, controlled, multi-armed, close-label, interventional study designed to demonstrate the efficacy of Ivermectin (oral/sub-cutaneous) with and without zinc in clearing the viral load of mild to moderate symptomatic COVID-19 patients.
Trial registration (2)	Clinical Trial registration will be done at www.clinicaltrials.gov
Protocol version (1)	Version 2.3
Sponsoring Body (1)	Smile Welfare Organization
Author details {5a}	Dr Sohaib Ashraf<br>Post-Graduate Cardiology Resident, Federal Post-Graduate Medical Institute, Lahore, Pakistan.
Name and contact information for the trial sponsor {5b}	Syed Kazam Ali, kazimformanite@gmail.com
Role of sponsor {5c}	There will not be any influential role of sponsor/funder in implication of trial, the study design, analysis of data and interpretation of final result outcomes.

Introduction

Background and rationale {6a}

As the world was still recovering from The Great War, a mayhem which shook the world and killed more than 40 million soldiers and civilians, mother earth suffered from a pandemic of Influenza. This deadly virus, in a year's span, spread everywhere on earth and contaminated roughly 500 million and killed 100 million people. Surprisingly after 101st year anniversary of that horrendous plague, we are again witnessing a fast spreading, highly infectious virus sweeping across the continents. This disease first originated in Wuhan, China in December 2019 which was later called as Corona Virus Disease -19 (COVID-19) or SARS-CoV-2. The illness was declared as pandemic by WHO in March 2020. Meanwhile, it had infected over 8.8 Million individuals and caused more than 465,000 deaths and still counting(3). Despite being huge data availability on the virus epidemiology, pathophysiology, virology, diagnosis, prevention and management still, till now all the explored options are not giving promising results with no established guidelines to treat this ailment and there's a need to find a cure for this deadly virus.

Despite of this wide range of options available for the treatment of COVID-19 none is proven completely effective against this virus and hence results of the clinical trials are controversial. This makes it the need of hour to think outside the box and prescribe newer formulations and conduct trails for treating COVID-19. This makes it the need of hour that we need to repurpose already FDA approved drug, Ivermectin, instead of making newer formulations. A novel idea of using micronutrients is also proposed as an anti-viral in this trial.

Recently a new FDA approved drug, Ivermectin, is being considered with promising results which has been a drug of prime focus in veterinary and human medicine for the control of parasitic infection and was the hub of attention in the 2015 Nobel Prize in Medicine. Ivermectin is a Nobel Prize winning FDA-approved broad spectrum anti-parasitic agent that has been repurposed for many non-parasitic diseases. Recently ivermectin has shown anti-viral properties against a broad range of RNA (DENV, West Nile Virus, Venezuelan equine encephalitis virus (VEEV) and influenza) and DNA viruses in vitro and in vivo. Primarily introduced as an inhibitor of interaction between the HIV-1 integrase protein (IN) and the importin (IMP) α/β1 heterodimer responsible for IN nuclear import, it has been established that ivermectin inhibits IN nuclear import and HIV-1 replication. Furthermore, anti-viral properties of ivermectin demonstrated that the virus inhibits nuclear import of host and viral proteins, including simian virus SV40, large tumor antigen (T-ag) and dengue virus non-structural protein(4). In 2014-2017, ivermectin was the focus of phase III clinical trial in Thailand against DENV infection, in which a single daily oral dose was observed to be safe and resulted in a significant reduction in serum levels of viral NS1 protein(5).

In the COVID-19 context, SARS-CoV-2, is a single stranded RNA virus that is closely related to SARS-CoV-1. Studies on SARS- CoV-1 proteins have revealed a potential role for IMPα/β1 during infection in signal-dependent nucleocytoplasmic shutting of its nucleocapsid protein (6, 7), that may impact host cell division (8). In addition, the SARS-CoV-1 accessory protein ORF6 has been shown to antagonize the antiviral activity of the STAT1 transcription factor by sequestering IMPα/β1 on the rough ER/Golgi membrane (9). Taken together, these reports suggested that ivermectin's nuclear transport inhibitory activity may be effective against SARS-CoV-2. Furthermore, ivermectin has also shown to act as an inhibitor of different cytokines including interleukin-6 (IL-6), one of the main causes of death (cytokine storm) due to COVID-19. In addition to this since ivermectin also induces autophagy through the Jak/Stat/PAK pathway. Since these pathways are involved in inducing expression of IL-6 and ivermectin induces

autophagy by inhibiting this pathway which might explain the possible mechanism how it inhibits different cytokines (immunomodulation).

Recently in an in-vitro study ivermectin inhibited the replication of SARS-CoV-2 at an IC50 of 2 µM which is ~50 fold its reported Cmax in the blood. The safety profile by FDA for human use has already been established for ivermectin against parasitic infections. Moreover, meta-analyses depict that ivermectin at high doses is safe (10, 11). The critical next step in further evaluation for possible benefit in COVID-19 patients will be to examine a multiple addition dosing regimen that mimics the current approved usage of ivermectin in humans. 338 clinical human trials have already been published on ivermectin worldwide and recently, subcutaneous efficacy of ivermectin in humans has also been shown (12-16)

Typical therapeutic doses of IVM are 150-200 μg/kg in humans while Cmax achieved for IVM are usually ~50 ng/ml. However, in some recent clinical studies IVM was used upto 800 ug/kg and the Cmax was ~100 ng/ml. If we dig into some historical data regarding IVM, we can see administrations of IVM up to 2 mg/kg are well tolerated(17). Furthermore, in another study, healthy volunteers who received 30-120 mg of IVM orally three times per week experienced no negative side-effects At these doses the Cmax of ivermectin was ~250 ng/ml which is still much lower than the IC50 reported against SARS-CoV-2. To this end, in an animal model the pulmonary concentration of ivermectin was shown to be 3-fold higher compared to the plasma. Historical data regarding ivermectin also indicates that administrations of ivermectin up to 2 mg/kg are well tolerated. In addition, many reports of ivermectin over dosage, without any side effects, also support the wide therapeutic window for this drug. The LD50 of IVM in different animals is 20-50 mg/kg(18), which is 250-fold more than its normal therapeutic dose (200 μg/kg).

The clinical trial conducted in Thailand against Dengue fever was given 400 μg/kg ivermectin once daily dose 3 times a week. This trial was based on the 25 μM IC50 of ivermectin against Dengue virus using vero cell line. Even though the trial did not show much improvement in terms of clinical efficacy, yet there were no side effects in terms of safety. In this context ivermectin's 10-fold lower IC50 values are promising against SARS-CoV-2 and warrant further investigation. To this end, some encouraging observations have been reported an observational case control study done on 14008 patients in 3 continents where ivermectin significantly reduced the mortality among the treated and nontreated groups (19).

No injectable human formulation of Ivermectin (sub-cutaneous) is worldwide available till now though studies have shown previously the use of subcutaneous formulation to be far more effective and safer (20). Various clinical interventional studies have been done on humans where injectable veterinary formulation was used to produce the desired effects in patients (12-16). For the purpose of research and considering the need of repurposing of drugs for COVID-19, patent veterinary subcutaneous ivermectin formulation will be injected to humans as subcutaneous injection for prolonged release and stable serum concentrations.

Another drug proposed to be administered is zinc. The rationale behind its usage is that the appropriate administration of Zinc supplement in sufficient therapeutic doses has a potential either to restore depleted immune cell function or to improve normal immune cell function. It may also act in a synergistic manner when co-administered with standard antiviral therapy (21). Antiviral properties of Zinc against a number of viral species are

mainly realized through the physical processes, such as virus attachment, infection, and uncoating, as well as through inhibition of viral protease and polymerase enzymatic processes (21).

Zinc is considered crucial for the proper folding and activity of various cellular enzymes and transcription factors and may be an important co-factor for numerous viral proteins as well. Zinc may interfere with the proteolytic processing of viral polyprotein by its misfolding, direct actions on the viral protease (as in picorna virus, encephalomyocarditis vvirus and polio virus) and alteration of the tertiary structure (as an encephalomyocarditis virus) (22). Zinc may also efficiently inhibit membrane fusion of respiratory syncytial virus, HSV, Semliki Forest virus and sindbis viruses, which is realized through binding to a specific histidine residue revealed on the viral E1 protein at low endosomal pH (23). Finally, Zinc have a potential for direct inactivation of the free Varicella-Zoster virus in vitro (24). Cell culture studies have demonstrated that high Zinc concentrations and the addition of pyrithione for stimulation of the cellular import of Zinc result in inhibition of the replication of various RNA viruses, including influenza virus, respiratory syncytial virus, and several picornaviruses (24, 25). It was suggested that in picornaviruses and coronavirus such an effect is realized due to the interference with viral polyprotein processing (26). Viral RNA-dependent RNA polymerase (RdRp) are suitable targets for novel antiviral drugs, since their activity is strictly virus-specific and may be blocked without severely affecting key cellular functions. Of note, an inhibitory effect of Zinc on function of viral RdRp was demonstrated in cases of rhinoviruses, HCV, and influenza virus (27, 28). In particular, in vitro studies have demonstrated that Zinc salts can reduce HCV replication in E. coli by 50% (at 100 µM ZincSO4) by inhibiting the HCV RdRp (29).

Nidoviruses is a large group of positive-strand RNA (+RNA) viruses, which includes major pathogens of humans and livestock, such as SARS-CoV and other human coronaviruses (2, 30). Zinc effectively inhibits the RNA-synthesizing activity of nidoviruses (including SARS-CoV) in vitro, which is realized through alteration of RdRp activity during the elongation phase of RNA synthesis, probably by directly affecting template binding (29). Thus, it may be suggested that in coronaviruses, Zinc 2+ may inhibit both the proper proteolytic processing of replicase polyproteins and RdRp activity (29). Of note, like other coronaviruses, SARS-CoV-2 causing COVID-19 also comes under nidovirus group. RdRp and 3CLpro protease of SARS-CoV-2 share over 95% of sequence similarity with those of SARS-CoV despite the fact that these two viruses demonstrate only 79% sequence similarity at the genome level (31)

The main foundation behind checking the combined efficacy is that ivermectin may act as a zinc ionophore which will allows zinc to enter the cells and inhibit SARS-CoV-2 replication. In addition to this zinc has shown allosteric modulation when combined with ivermectin to activate P2X4 receptors (32). Since both ivermectin and zinc are considered potential drug targets against SARS-CoV-2. And research has demonstrated a potentiation effect of ivermectin and zinc combinations which may be a novel strategy to develop more effective approaches in treating COVID-19. Thus, a supplementation of zinc along with ivermectin may facilitate each other's contribution in a better therapeutic outcome.

Objectives {7}

The objective of the study is to measure the efficacy of Ivermectin (oral/sub-cutaneous) in reducing the severity of symptoms, length of hospital stay along with earlier qRT-PCR clearance and radiologically better HRCT chest as compared to control group. Additional observation will include prevention of onset of pneumonia and declined mortality rates as compared to standard treatment for mild to moderate COVID-19 patients. Ivermectin will also be tested in combination with zinc.

Trial design {8}

This is a placebo-controlled, multi-armed, add-on, interventional, randomized trial using parallel group design. It is a close-labeled and adaptive design with a superiority framework.

Methods: Participants, interventions and outcomes

Study setting {9}

Clinical sampling will be done from Shaikh Zayed Post-Graduate Medical Complex, Doctors' Lounge and Ali Clinic.

Eligibility criteria {10}

All diagnosed COVID-19 patients with a mild to moderate disease will be screened.

Inclusion Criteria:

- Positive RT-PCR (Real Time Polymerase Chain Reaction)
- Both genders and age 18 years and above

Exclusion Criteria:

- Presence of any co-morbidities like liver disease, thyroid dysfunction, ischemic heart disease, immuno-compromised patient or any other chronic ailment (other than Diabetic and Hypertensive)
- Females who are pregnant and breast feeding
- If a patient is allergic to either Ivermectin or Zinc.

Who will take informed consent? {26a}

Site investigator will take written informed consent from all trial participants by giving them the specifically constructed informed consent form (provided at the end of proposal)

Additional consent provisions for collection and use of participant data and biological specimens {26b}

Site investigators will also be responsible for taking consent regarding every other matter if required. Informed consent form will contain section on permission to draw and conduct specified tests on blood samples and conduct radiological investigations as per protocol of the study.

Interventions

Explanation for the choice of comparators {6b}

All 6 arms will be receiving standard care as per protocol of the study setting. Placebo comparator group, arm X, will receive placebo i.e subcutaneous normal saline and empty capsule, in addition to standard care.

Interventional arms will be given treatment regimen as follows along with standard care.

- Arm A will be receiving Ivermectin (subcutaneous)
- Arm B will be receiving Ivermectin (subcutaneous) with Zinc (oral)
- Arm C will be receiving Ivermectin (oral)
- Arm D will be receiving Ivermectin (oral) with Zinc (oral)
- Arm E will be receiving Zinc (oral)
- Arm X will be placebo-control group

Intervention description {11a}

Oral or injectable Ivermectin and oral Zinc Sulphate (tablet) will be obtained from pharmacy.

- Arm A will receive IVM subcutaneously at 200 μg/kg/48 h with placebo (empty capsule).
- Arm B will receive IVM subcutaneously at 200 μg/kg/48 h along with Zn Sulphate (oral capsule) 20mg/every eight hours.
- Arm C will be given IVM orally at 0.2 mg/kg/day with placebo (empty capsule).
- Arm D will be given IVM orally at 0.2 mg/kg/day along with Zn Sulphate (oral capsule) 20mg/every eight hours.
- Arm E will only receive Zn Sulphate (oral capsule) 20mg/every eight hours with placebo (subcutaneous normal saline 2ml).
- Arm X will receive placebo i.e subcutaneous normal saline 2ml 48 hourly and empty capsule 8 hourly.

All 6 arms will be receiving standard care as per protocol and COVID-19 guidelines of the study setting.

Criteria for discontinuing or modifying allocated interventions {11b}

Fixed dose will be given throughout the study and interventional drug administration will be stopped immediately in following conditions:

1. Patient becomes symptomatic during trial conduction
2. Any adverse drug reaction
3. Organ failure secondary to any administered drug
4. Patient denies/back off from the further participation

Regardless of any of the condition, participant's data will be retained and analyzed in the trial in order to follow-up and prevent any data missing.

Strategies to improve adherence to interventions {11c}

To improve adherence to the intervention, participants will be counselled about the advantages of this study. All the participants will be monitored regarding compliance to their assigned treatment strategy and health professionals will give drugs. As this trial will include admitted patients from quarantine centres of Pakistan direct observational method will be sufficient to make sure compliance and adherence to the interventions.

Relevant concomitant care permitted or prohibited during the trial {11d}

As per hospital protocol (study setting), the care and interventions permitted will be used and no specific prohibited care is in this trial. As far as drug reactions are concerned, they will be treated by health care workers on the spot and will be reported afterwards.

Provisions for post-trial care {30}

No post-trial care will be needed in our study setup as half-life of administered drugs is within hours to days.

Outcomes {12}

Primary Outcomes

- Time Taken for Alleviation of Symptoms
- Viral Load Clearance
- HRCT Chest Score
- Clinical Grading Score (CGS) at Day 6

Secondary Outcomes:

- 30 Day Mortality

Additional Outcomes:

- Median time to clinical improvement of clinical grade score
- Median time to clinical improvement of severity of symptoms
- Median time to clinical improvement of degree of fever
- Median time to clinical improvement of cough
- Median time to clinical improvement of shortness of breath
- Median time to clinical improvement of myalgia
- Median time to clinical improvement of “how sick do you feel”

Operational Definitions

Severity of respiratory symptoms is classified as mild, moderate, severe and Acute Respiratory Distress Syndrome ARDS(45)

Mild: "Symptoms of an upper respiratory tract viral infection i.e. low-grade fever, dry cough, sore throat, nasal congestion, malaise."

Moderate: "Respiratory symptoms (cough and shortness of breath) without signs of severe pneumonia."

Severe: "Fever is associated with severe dyspnoea, respiratory distress, tachypnoea (> 30 breaths/min), and hypoxia (SpO2 < 90% on room air)"

Viral Load Clearance will be done on admission day (0 day) and then tested second time for SARS-CoV-2 by RT-PCR at day 7.

Duration of hospital stay

The number of days the patient stayed in the hospital setting during course of his treatment. The date of admission and date of discharge would give us the outcome in terms of good or poor prognosis.

HRCT chest score

High resolution computed tomography (HRCT) chest scoring will be done to access the lung involvement on day 6.

HRCT SCORING will be done as follows: Lungs divided into five lobes and each lobe is given 1 number. Total score is 25. Each lobe is scored from 0 to 5 as:

- 0 = no involvement
- 1 = <5% involvement
- 2 = 25% involvement
- 3 = 26%-49% involvement
- 4 = 50%-75% involvement
- 5 = >75% involvement

The individual lobar scores i.e. from 0 (no involvement) to 25 (maximum involvement) (39) make up the total HRCT chest score.

The HRCT findings are described via standard international terms, which are classified by the Fleischner Society glossary with peer-reviewed literature on viral pneumonia. The terms being used are ground glass opacity (GGO), crazy-paving pattern, and consolidation (43, 44).

Fever will be categorized as no fever (98-99 °F), mild (>99-<100 °F), moderate (100-101.9 °F) and severe (≤102°F).

Cough will be graded as mild (occasional but transient cough), moderate (recurrent but slightly affecting daily activities) and severe (recurrent cough but significantly limiting daily activities).

Myalgia/How sick do you feel will be subjective feelings evaluated on a 10-point scale and classified as mild (1-3), moderate (4-6) and severe (7-10).

Shortness of breath will be classified

Grade 1: Short of breath only on strenuous exercise

Grade 2: Breathlessness when walking upstairs

Grade 3: Breathlessness when walking on flat surface

Grade 4: Breathlessness with talking

Grade 5: Too breathless at rest.

Grading of Clinical Status:

Grading of clinical status will be assessed using following criteria:

Grade 0; Not hospitalized and capable enough to resume normal daily life activities

Grade 1: Not hospitalized but incapable of resume normal daily life activities

Grade 2: Hospitalized but do not require supplemental oxygen therapy

Grade 3: Hospitalized but require supplemental oxygen therapy

Grade 4: Hospitalized, requiring mechanical ventilation

Grade 5: Hospitalized, requiring nasal high-flow supplemental oxygen and/or noninvasive mechanical ventilation

Grade 6: Hospitalized, requiring invasive mechanical ventilation

Grade 7: Death

Participant timeline {13}

Time schedule of enrolment, interventions (including any run-ins and washouts), assessments, and visits for participants has been shown in a schematic diagram.

Sample size {14}

30 patients in each arm with a total of 180 patients sample size.

Recruitment {15}

Recruitment will be done in multiple designated corona centres by visiting COVID-19 wards for mild to moderate symptomatic patients. All patients' record will be analyzed at the center and eligible participants will be separated. All the eligible participants will be assessed according to our inclusion and exclusion criteria. Fully equipped site investigators with full precautions will do all of these proceedings. At the start of study trial, all recruited participants have to provide written informed consent form as per plan in order to get them enrolled for the study.

Assignment of interventions: allocation

Sequence generation {16a}

Stratification for initial covid-19 status (or days from initial symptoms as a proxy), age groups, gender and co-morbidities will be used to ensure that groups remain balanced in size for either arm after written informed consent to participate in our study. Randomization will be done using lottery method. As patients might be admitted at different times so they will be recruited after taking written informed consent (following all standard protocol for infection control and disinfection) and will be randomized by selecting a slip from the box containing 30 slips of each arm labeled as A, B, C, D, E and X. Arm A, B, C, D and E will be the add-on interventional arms while arm X will be the placebo arm.

Concealment mechanism {16b}

The allocation sequence will be computer generated that will be concealed from all site investigators, allocated participants and treatment providers until the final interventional allocation is done.

Implementation {16c}

The site investigators who will do the recruitment of interventional groups will request the principal investigator, Shoaib Ashraf (ShA) for randomization. The principal investigator (ShA) will send his answer form to the treatment providers in concealed envelops at allocated corona centres. The therapists will have no influential role in study outcomes and analysis while only disclosing the treatment plans to patients. Site investigators and other study members that are involved in participant’s enrolment will not be allowed to receive allocation information in order to prevent study bias.

Assignment of interventions: Blinding

Who will be blinded {17a}

Trial participants, care providers, outcome assessors and data analysts are blinded, respectively, by using placebo group, by using site investigators to provide placebo or drugs to participants, by using blinded clinicians to assess the clinical outcome and laboratory or radiological findings while by using analysts from other institution that are not having any conflict of interest in research while study chair being the only person knowing the participants allocated and analyzed in interventional arm.

This study is quadruple blinded in which experimental drug and placebo will look alike, as they will have same packing but unique randomization codes. Participants, site investigators, care providers, outcome assessors, study coordinators, data managers, and statisticians will be blinded, and blinding codes will be revealed at the end of this study.

Procedure for unblinding if needed {17b}

Unblinding is permissible if the patient develops symptoms and needed extra treatment by revealing a participant's allocated intervention during the trial. If unblinding is required, the trial managers and data coordinators will have access to group allocations and any unblinding will be reported.

Data collection and management

Plans for assessment and collection of outcomes {18a}

Microsoft Access, a database management system (DBMS) of Microsoft Office will be used to ensure the data safety. Two site investigators will enter the data, recheck twice for possible errors separately, and make certain its integrity. Principal investigators will visit twice weekly the study site while ethical committee will overview the study on weekly basis. Trial steering committee members will make unexpected and unplanned visits as well. There is no conflict of financial and non-financial interest with sponsors and researchers.

Plans to promote participant retention and complete follow-up {18b}

All the participants will be ensured their safety and will be guided about the study conduction and its beneficial outcomes. The study will be conducted while participants being admitted in corona centres during 14 days of their disease course or till the RT-PCR SARS-CoV-2 becomes negative. All the data will be collected while the patient is already admitted in the hospital except 30-day mortality. Contact numbers and addresses of all participants will be reported at the start of study for which can be used, if needed, after written consent from the patient. Follow up will be done using phone numbers of the patients to access the mortality benefit of intervention.

Data management {19}

Participants IDs will be used for confidentiality purposes and these IDs will be linked to demographic information securely and separately. The final data set of RCT will have coded data and can only be assessed by principal investigators. All outcomes will be double-checked by the researchers prior to data collection and data storage. To ensure data's integrity and safety various meetings by the research team will be conducted on regular basis.

Confidentiality {27}

In addition, confidentiality of participants' data is ensured by using participants' IDs rather than identifiable information in the dataset (i.e. coding) and by storing the document linking the IDs to the identifiable information separately and securely.

Plans for collection, laboratory evaluation and storage of biological specimens for genetic or molecular analysis in this trial/future use {33}

Trained staff will collect nasopharyngeal swabs samples as per biosafety and personal safety guidelines of World Health Organization. Samples will be maintained at -80 degrees. The investigators will monitor variables including quality of life, symptom scores, duration stayed, oxygenated and ventilated along with number of hospitals stayed days and mortality. Patients will be evaluated clinically on daily basis; relevant investigations will be repeated based on following plan.

Statistical methods

Statistical methods for primary, secondary and additional outcomes {20a}

Mean ± S.D will be used for quantitative data and f(%) will be used for categorical data. Frequency and percentages will be measured for categorical data. Data normality will be checked using Shapiro Wilks test., if data is normal independent sample t-test will be used to compare quantitative outcome such as mean hospital stay otherwise Mann Whitney U test will be used to compare median of these quantitative data. Chi-square t-test/ Fisher's Exact test will be applied to compare severity of symptoms and outcome (discharge or mortality), etc. For follow up analysis Wilcoxon test will be applied. In univariate analyses, we will apply a log-rank test to compare time taken for symptoms' alleviation, time to improvement in clinical grade score, severity of symptoms, degree of fever, cough, shortness of breath, myalgia and "how sick do you feel". We will apply the Fisher's Exact test to evaluate viral clearance, HRCT score at day 6 and 30-day mortality. Multivariate regression models will be applied to adjust for the effects of age (<50 or >=50), gender, baseline CGS and co-morbidities. Kaplan Meier method will be used to plot survival curves for time to symptoms' alleviation. Ordinal logistic regression models will be applied for the multivariate analyses of ordinal outcomes considering proportional odds. P-value ≤ 0.05 will be considered as significant. Data will be analyzed using SAS version 9.4.

Interim analyses {21b}

The risk aptitude for this study is customized from medium to high risk, considering the use of animal formulation of injectable ivermectin and iodine complex. Along with that, as it involves vulnerable COVID-19 patients and novel drugs are being tested for repurposing strict safety measurements will be taken. As a part of our safety measurements the co-investigator, Dr Uzma Nasim Siddique (Assistant Professor Medicine, SZH, Lahore), under the supervision of the biostatistician, Prof. Dr. Muhammad Azam (Dean faculty of biostatistics, UVAS, Lahore) will conduct an interim analysis. The prime focus of this analysis will be mortality and incidence of any serious adverse effects. Two interim analyses are planned, first after completion of the study on 60 subjects and then after 120 subjects. For these specific outcomes, those conducting the interim analysis will be unblinded. No stopping rules for the primary endpoint have been defined as this is the first trial of its kind.

Analysis will be conducted between control and treatment group. The clinical outcome comparison for these two groups will be obtained using the two-group chi-square test or Fisher exact test, where applicable. The trial will immediately be stopped, and relevant and appropriate authorities shall be consulted if the difference between the groups is significant enough to give a $p < 0.01$.

Methods for additional analyses (e.g. subgroup analyses) {20b}

Adjusted and subgroups analysis may be applied as per the biostatistician, if needed. In that case both unadjusted and adjusted analyses are provided along with the main analysis

Methods in analysis to handle protocol non-adherence and any statistical methods to handle missing data {20c}

The intention-to-treat analysis set will be used to test the superiority. All patients will be considered as randomized despite receiving the randomized treatment as per our anticipation. Reasons for each group's randomization and withdrawal will be reported and compared qualitatively and sensitivity analysis (augmented data) is being used to overcome the effect of any missing data on results. The participants who withdraw consent for continued follow-up (Dropouts) will be assessed by modern imputation methods for missing data

Plans to give access to the full protocol, participant level-data and statistical code {31c}

Principal investigators will have access to the full trial dataset in order to ensure that the overall results are not disclosed by an individual of trial prior to the main publication. Grant public access to the full protocol, participant-level dataset, and statistical code will be given through clinicaltrials.gov.

Oversight and monitoring

Composition of the coordinating centre and trial steering committee {5d}

Coordinating center and trial steering committee will be comprised of medical lab technologist (Dr. Sidra Ashraf, PhD), clinical pharmacist (Dr Faisal Nadeem, PhD), clinical pharmacologists and toxicologist (Dr. Moneeb Ashraf, PhD), virologist (Prof. Dr. Mateen Izhar, PhD), immunologist (Prof. Dr. Ali Ahmad), biostatisticians (Prof. Dr. Muhammad Azam, PhD), public health expert (Prof. Dr. Usman Iqbal, PhD), epidemiologist (Prof. Dr. Ayesha Humayun), ethical expert (Prof. Dr. Muhammad Suhail) and consultants of medicine (Prof. Dr. Uzma Nasim Siddique, FCPS medicine), pulmonology (Prof. Dr. Talha Mahmud, MD Pulmonology) and cardiology (Prof. Dr. Amber Malik, MRCP) department. This committee will be responsible for the safety, trial safety and dosage calculation to get results of endpoints. This will have all the authority to stop the clinical trial all together. Data management team will be comprised of principal investigators, co-investigators and site investigators. This team will be responsible to ensure the execution of the clinical trial in best possible way as defined by the study protocol. Site investigators are responsible for data collection and quality check of data at collection points/ study setting. Principal Investigators, Dr. Sohaib Ashraf (SoA) will manage data compilation, cleaning, editing, and entry along with biostatistician.

Composition of the data monitoring committee, its role and reporting structure {21a}

Data monitoring committee (DMC) has a biostatistician (Prof. Dr. Muhammad Azam, PhD), an epidemiologist (Prof. Dr. Ayesha Humayun, PhD), and public health expert (Prof. Dr. Usman Iqbal, PhD), a microbiologist (Prof. Dr. Mateen Izhar), a pulmonologist (Prof. Dr. Talha Mehmood) and principal investigators (Prof. Dr. Muhammad Ashraf, PhD and Dr. Shoaib Ashraf, PhD) in it. There is no financial or non-financial conflict of interest as the committee will be independent of sponsor and competing interests.

Adverse event reporting and harms {22}

The researchers will record any adverse, unpredictable or undesirable sign and symptom and it will be discussed with the care providers. A comprehensive evaluation will be conducted to evaluate the co-relation between experimental drug and the developing signs and symptoms. The investigator will respond appropriately to ensure the wellbeing of the patient in case of any unforeseen event and all the details will be written carefully. Moreover, regular follow-up will be made certain until the patient regains his/her health. If the adverse event happens during the study intervention will be reported Institutional Review Board (IRB).

Frequency and plans for auditing trial conduct {23}

Weekly audit will be done by principal investigators. Monitors will audit by visiting trial sites while performing and resolving solutions to various problems. The monitor will verify the following variables for all patients on every visit: Bio data of participants, signed informed consent, eligibility criteria, date of randomization, group allocation, treatment assigned and adverse events if any. Auditing of the clinical trial will be done by trail steering committee in weekly zoom meeting where all the audits will be provided by the site investigators and research coordinators.

Plans for communicating important protocol amendments to relevant parties (e.g. trial participants, ethical committees) {25}

In order to modify protocols including eligibility criteria and outcomes, permission will be needed to get approved by the trial steering committee and the notification will be done to relevant parties including IRB trial registries and journals. All the plans about any amendments in our trial will be communicated to the trial site staff in person.

Dissemination plans {31a}

The publications subcommittee will review the publication, all the endpoint data, primary outcome analysis and the study results and recommend the changes to the author. After the changes being done it will finally submit its recommendations to the steering committee for approval. Study results will be disclosed to all study participants, member physicians, patients and other medical personnel.

Discussion

The study will be limited to viral load clearance at day 7 rather than measuring viral load reduction.

Abbreviations

Abbreviation	Full Form
BSL-3	Bio safety laboratory level 3
COVID-19	Corona Virus Disease 19
DENV	Dengue virus
DM	Diabetes Mellitus
DNA	Deoxyribose Nucleic Acid
FAIR	Findable, Accessible, Interoperable, Re-usable
FDA	Food and Drug Administration
HIV	Human Immunodeficiency Virus
HRCT	High Resolution Computerized Tomography
HTN	Hypertension
IC50	Inhibitory Concentration 50
IRB	Institutional Review Board
IVM	Ivermectin
LD50	Lethal Dose 50
qRT-PCR	Qualitative real time polymerase chain reaction
RNA	Ribose Nucleic Acid
SARS-CoV-1	Severe acute respiratory syndrome coronavirus 1
SARS-CoV-2	Severe acute respiratory syndrome coronavirus 2
SZH	Shaikh Zayed Hospital
UVAS	University of Veterinary & Animal Sciences
VEEV	Venezuelan equine encephalitis virus

Declarations

Acknowledgements:

The authors would like to show gratitude to the following colleagues who provided moral support and intellectual inputs in designing this trial: Dr. Asad-ullah- Assistant Director Drug Regulation Authority Pakistan, Dr. Zaka-ur-Rehman, PhD- Chief Drug Controller Punjab, Prof. Dr. Xin Zhao, PhD James McGill Professor, McGill University, Sainte-Anne-de-Bellevue, Quebec, Canada and Prof. Dr.Roger Prichard,PhD, McGill Professor of Parasitology & Pharmacology, Institute of Parasitology, McGill University, Sainte-Anne-de-Bellevue, Quebec, Canada.

Funding {4}

Funding will be provided by Smile Welfare Organization, Pakistan.

Availability of data and materials {29}

The datasets used or analysed during the current study will be available from the corresponding author upon reasonable request.

Ethics approval and consent to participate {24}

Ethical approval has been applied for authorization to institutional review board of Shaikh Zayed Hospital, Lahore, PK. Written, informed consent to participate will be obtained from all participants.

Competing interests {28}

The authors affirm that they have no competing interests and according to the standards of scientific integrity the publication of both positive and negative study results will be ensured. All the study data (if reasonable) will be made accessible guided by the FAIR principles along with the perspective of relevant laws and privacy regulations. Authorship eligibility follows conventional academic standards. No professional writers had been involved in this.

Figure 1: CONSORT 2010 Flow Diagram

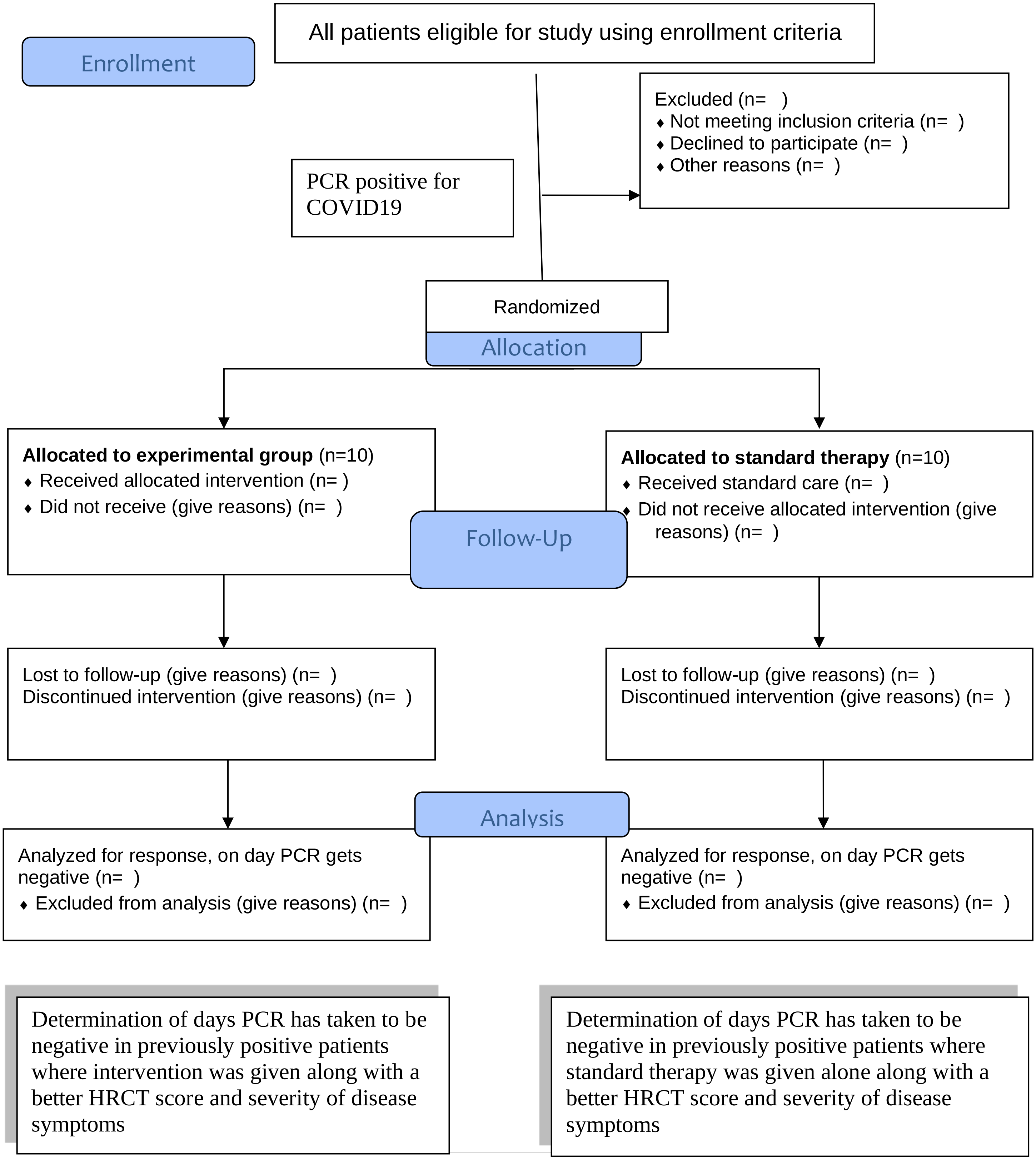

**FIGURE 2**	***STUDY PERIOD***															
	**Enrolment Allocation**	**Post-allocation**														Follow-up
TIME POINT	Day 0	Day 1	Day 2	Day 3	Day 4	Day 5	Day 6	Day 7	Day 8	Day 9	Day 10	Day 11	Day 12	Day 13	Day 14	Day 30
ENROLMENT:																
Eligibility screen Randomization	x															
Informed consent	x															
Demographics [1]	x															
Medical history and epidemiological information [2]	x															
INTERVENTIONS:																
Pharmaceutical Agent(s)		x	x	x	x	x	x	x	x	x	x	x	x	x	x	
Placebo		x	x	x	x	x	x	x	x	x	x	x	x	x	x	
ASSESSMENTS:																
Physical exam [3]	x															
Vital signs [4]	x															
CBC [5]	x															
LFTs [5]	x															
RFTs [5]	x															
Clinical evaluation	x															
12 lead ECG	x															
Chest X-ray	x															
Alleviation of symptoms		x	x	x	x	x	x	x	x	x	x	x	x	x	x	
RT-PCR [6]								x								
HRCT Chest [7]							x									
Clinical Grading Status on 7-pointer scale	x		x		x		x		x		x		x			
Fever	x	x	x	x	x	x	x	x	x	x	x	x	x	x	x	
Cough	x	x	x	x	x	x	x	x	x	x	x	x	x	x	x	
Shortness of breath	x	x	x	x	x	x	x	x	x	x	x	x	x	x	x	
Myalgia	x	x	x	x	x	x	x	x	x	x	x	x	x	x	x	
Rate your emotional status	x	x	x	x	x	x	x	x	x	x	x	x	x	x	x	
How sick do you feel	x	x	x	x	x	x	x	x	x	x	x	x	x	x	x	
Oxygen saturation on room air	x	x	x	x	x	x	x	x	x	x	x	x	x	x	x	
Oxygen Therapy	x	x	x	x	x	x	x	x	x	x	x	x	x	x	x	
Adverse effects of drug administration	x	x	x	x	x	x	x	x	x	x	x	x	x	x	x	
Mortality																x

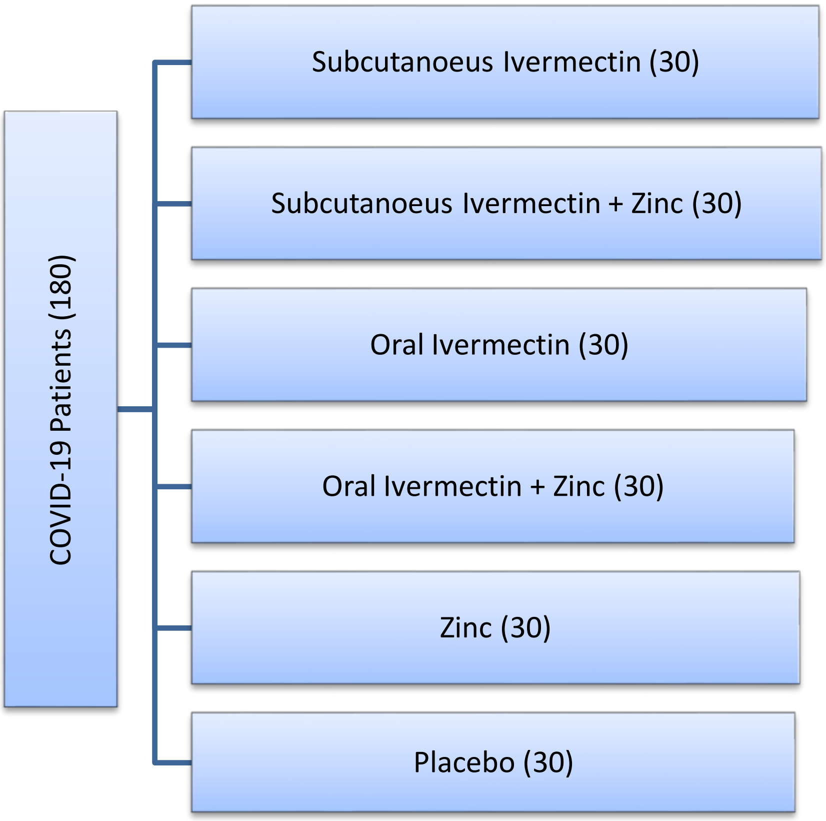

FIGURE 3 : Distribution of patients in multiple arms

Informed Consent Form {32}

This consent form addresses the participants of the clinical trial.

The title of our research project is: Efficacy of Subcutaneous Ivermectin with or without Zinc in Mild to Moderate COVID-19 patients (SIZI-COVID-PK)

Name of Principal Investigator: Dr. Sohaib Ashraf
Name of Organization: Federal Post Graduate Medical Institute, Shaikh Zayed Medical Complex Lahore

This Informed Consent Form has two parts:
- Information Sheet (to share information about the research with you)
- Certificate of Consent (for signatures if you agree to take part)

PART I:

Introduction:
I am ______________________ Our team is conducting a research on COVID-19, which is a global health concern. I am providing you with all the necessary information and invite you to be part of this research. Before making any decision, whether you will participate or not, you may take your time and talk to someone you feel comfortable with about the research. If you have any difficulty in understanding anything, you may stop me as I go through the information and I will take time to explain. If you have questions later, you can ask them of me, the study doctor or the staff. A copy of the full informed consent form will also be provided to you.

EXPLANATION:
The proposed study is a placebo-controlled, add-on, randomized trial using parallel group designs. This is a close-label and adaptive design with 1:1 allocation ratio and superiority framework. It will be conducted Shaikh Zayed Hospital. This study will have five arms (10 patients in each): interventional arm A will be Ivermectin alone, arm B will be Ivermectin with Zinc, arm C will be encapsulated Iodine complex, arm D will be Iodine complex (syrup form) (as add-on) while arm X will be standard care with placebo. Data will be collected on self-constructed, close-ended questionnaires after obtaining written consent. Data will be analyzed using SPSS version 25. COVID-19 patients will be monitored by qRT-PCR and HRCT (High Resolution Computed Tomography) chest. In addition to these clinical improvements, duration of hospital stay, and mortality benefits will be analyzed in both groups. The trial will aid in devising a better strategy to cope COVID-19 in a relatively inexpensive and accessible. The implications are global, and this could prove itself to be the most manageable intervention against COVID-19 especially for patients from limited-resource countries with deprived socioeconomic statuses.

VOLUNTARY PARTICIPATION:
It's a voluntary participation in this trial. Whether you choose to be a part of it or not, all the services you receive at this hospital will continue and nothing will change. If you choose to participate you will have the authority to change your mind later and stop participating even if you agreed earlier.

PROCEDURES AND PROTOCOL
Participants in one group will be given the experimental treatment along with the standard treatment while participants in the other group will only be given the standard treatment as per hospital protocol. The healthcare workers will be monitoring you and the other volunteers'

vigilantly during the study. If there is anything you are anxious about or that is troubling you about the research, please talk to me or one of the other colleagues. For the purpose of this study, we will be with drawing 10ml of your blood (when needed), which will help us access your clinical laboratory data. Blood will be withdrawn from your arm, via trained staff, using a syringe through arterial/venous site.

You will not be given any monetarily benefit to take part in this research and confidentiality will be maintained. Identity of those taking part in this trial will not be revealed. The personal information that we collect from this research project will be kept confidential. Your personal information will be given numbers instead of your names. Only the researchers will be aware of the assigned number and we will lock that information up with a lock and key. This information can only be accessed by study director and study chair.

SHARING THE RESULTS:

Prior to making the knowledge, we get from this research, publically available to the outside world, It will be shared with the participants through community/zoom meetings. Confidential information will not be shared. After these meetings, we will publish the results so that the knowledge gained can be shared with rest of the world.

RIGHT TO REFUSE OR WITHDRAW:

You do not have to take part in this research if you do not wish to do so. You may also stop participating in the research at any time you choose. It is your choice, and all of your rights will still be respected. Alternatives to Participating If you do not wish to take part in the research, you will be provided with the established standard treatment available at the center/institute/hospital.

Who to Contact:

If you have any questions, you may ask them now or later, even after the study has started. If you wish to ask questions later, you may contact any of the following:

Dr. Sohaib Ashraf +923334474523
Dr Ahmad Imran +923338110708
Dr Uzma Mamoon +923002102436
Dr Moneeb Ashraf +923334461038

This proposal has been reviewed and approved by ethical review board of Shaikh Zayed medical complex, Lahore, which is a committee whose task it is to make sure that research participants are protected from harm. You can ask me any more questions about any part of the research study, if you wish to. Do you have any questions?

PART II: Certificate of Consent

I have read the foregoing information, or it has been read to me. I have had the opportunity to ask questions about it and any questions that I have asked have been answered to my satisfaction. I consent voluntarily to participate as a participant in this research.

Print Name of Participant__________________
Signature of Participant ___________________
Date ___________________________ Day/month/year

If Illiterate (consent form being read to the witness)
I have witnessed the accurate reading of the consent form to the potential participant, and the individual has had the opportunity to ask questions. I confirm that the individual has given consent freely.

Print name of witness_____________________
Thumb print of participant/ Signature of witness ______________________
Date ________________________ Day/month/year

I have accurately read out the information sheet to the potential participant, and to the best of my ability made sure that the participant understands.
I confirm that the participant was given an opportunity to ask questions about the study, and all the questions asked by the participant have been answered correctly and to the best of my ability. I confirm that the individual has not been coerced into giving consent, and the consent has been given freely and voluntarily.

A copy of this ICF has been provided to the participant.

Name of the person taking the consent ________________________
Signature of Researcher /person taking the consent__________________________
Date ___________________________ Day/month/year

PERFORMA

Medical Record #: ____________ ID: ___________

Name (optional): _____________ Age: _______ Gender: M / F

Domicile: ___________________ Oxygen Saturation: ___________

Study Center: ____________ Reference Doctor: _________

Contact Number: ________ Profession: _____________

Contact History: ______________ Blood Group: _____

Fever: __________ Fatigue: _______ Dry Cough: __________

SOB: ___________ Anorexia: ______ Sputum production: ____

Pharyngitis: ______ Myalgia: _______ GI disturbances: _______

Severity of Symptoms: ____________ End Result: ____________________

CBC: ____

RFTs: ____

LFTs: ____

ECG: ____

Chest X-ray: ____

Clinical Course of Treatment:

____

____

___

Clinical Data:

____

____

___

Day of enrollment: __________ Date: _________ ID: _________

Please rate the average severity of your corona symptoms over the last 24 hours for each symptom:

**Symptom**	Not Sick	Very Mild	Mild	Moderate	Severe
Fever					
Cough					
Sputum					
Headache					
Sneezing					
Aches & Pains					
Nausea					
Fatigue					
Running Nose					
Sore Throat					
Chills & Rigors					
Shortness of Breath					
GI disturbance					
Loss of smell					
Change of taste					
Insomnia					
Malaise					

Rate your emotional wellness out of 10 ____ (1 being severely depressed, 10 being perfectly health)

PCR at Day 7 : ________

Grading:

Choose the appropriate.

Grade	Day 0	Day 2	Day 4	Day 6	Day 8	Day 10	Day 12
Not hospitalized with resumption of normal activities (Grade 1)							
Not hospitalized, but unable to resume normal activities (Grade 2)							
Hospitalized, not requiring supplemental oxygen (Grade 3)							
Hospitalized, requiring low flow supplemental oxygen (Grade 4)							
Hospitalized, requiring high flow (Grade 5)							
Hospitalized, requiring mechanical ventilation (Grade 6)							
Death (Grade 7)							

REFERENCES :

1. Amit N. Patel MD M, 2; Sapan S. Desai MD PhD MBA3; David W. Grainger PhD1; Mandeep R. Mehra,MD, MSc4. Usefulness of Ivermectin in COVID-19 Illness.
2. Gorbalenya AE, Enjuanes L, Ziebuhr J, Snijder EJ. Nidovirales: evolving the largest RNA virus genome. Virus research. 2006;117(1):17-37.
3. Organization WH. Coronavirus disease 2019 (COVID-19): situation report, 72. 2020.
4. Forwood JK, Jans DA. Nuclear Import Pathway of the Telomere Elongation Supressor TRF1: Inhibition by Importin α. Biochemistry. 2002;41(30):9333-40.
5. Caly L, Druce JD, Catton MG, Jans DA, Wagstaff KM. The FDA-approved Drug Ivermectin inhibits the replication of SARS-CoV-2 in vitro. Antiviral research. 2020:104787.
6. Rowland RR, Chauhan V, Fang Y, Pekosz A, Kerrigan M, Burton MD. Intracellular localization of the severe acute respiratory syndrome coronavirus nucleocapsid protein: absence of nucleolar accumulation during infection and after expression as a recombinant protein in Vero cells. Journal of virology. 2005;79(17):11507-12.
7. Timani KA, Liao Q, Ye L, Zeng Y, Liu J, Zheng Y, et al. Nuclear/nucleolar localization properties of C-terminal nucleocapsid protein of SARS coronavirus. Virus research. 2005;114(1-2):23-34.
8. Hiscox JA, Wurm T, Wilson L, Britton P, Cavanagh D, Brooks G. The coronavirus infectious bronchitis virus nucleoprotein localizes to the nucleolus. Journal of Virology. 2001;75(1):506-12.
9. Frieman M, Yount B, Heise M, Kopecky-Bromberg SA, Palese P, Baric RS. Severe acute respiratory syndrome coronavirus ORF6 antagonizes STAT1 function by sequestering nuclear import factors on the rough endoplasmic reticulum/Golgi membrane. Journal of virology. 2007;81(18):9812-24.
10. Navarro M, Camprubí D, Requena-Méndez A, Buonfrate D, Giorli G, Kamgno J, et al. Safety of high-dose ivermectin: a systematic review and meta-analysis. Journal of Antimicrobial Chemotherapy. 2020;75(4):827-34.
11. Nicolas P, Maia MF, Bassat Q, Kobylinski KC, Monteiro W, Rabinovich NR, et al. Safety of oral ivermectin during pregnancy: a systematic review and meta-analysis. The Lancet Global Health. 2020;8(1):e92-e100.
12. Zeitler K, Jariwala R, Restrepo-Jaramillo R, Kapadia S, Casanas B, Alrabaa S, et al. Successful use of subcutaneous ivermectin for the treatment of Strongyloides stercoralis hyperinfection in the setting of small bowel obstruction and paralytic ileus in the immunocompromised population. Case Reports. 2018;2018:bcr-2017-223138.
13. Marty FM, Lowry CM, Rodriguez M, Milner DA, Pieciak WS, Sinha A, et al. Treatment of human disseminated strongyloidiasis with a parenteral veterinary formulation of ivermectin. Clinical Infectious Diseases. 2005;41(1):e5-e8.
14. Roberge C, Cros J-M, Serindoux J, Cagnon M-E, Samuel R, Vrlinic T, et al. BEPO®: Bioresorbable diblock mPEG-PDLLA and triblock PDLLA-PEG-PDLLA based in situ forming depots with flexible drug delivery kinetics modulation. Journal of Controlled Release. 2020.
15. Barrett J, Broderick C, Soulsby H, Wade P, Newsholme W. Subcutaneous ivermectin use in the treatment of severe Strongyloides stercoralis infection: two case reports and a discussion of the literature. Journal of Antimicrobial Chemotherapy. 2016;71(1):220-5.
16. Pacanowski J, DOS SANTOS M, Roux A, LE MAIGNAN C, Guillot J, Lavarde V, et al. Subcutaneous ivermectin as a safe salvage therapy in Strongyloides stercoralis hyperinfection syndrome: a case report. The American journal of tropical medicine and hygiene. 2005;73(1):122-4.

17. Costa JL, Diazgranados JA. Treatment of human diseases involving dysregulation or dysfunction of the nervous system. Google Patents; 1994.
18. Dadarkar S, Deore M, Gatne M. Comparative evaluation of acute toxicity of ivermectin by two methods after single subcutaneous administration in rats. Regulatory Toxicology and Pharmacology. 2007;47(3):257-60.
19. Patel A. Usefulness of Ivermectin in COVID-19 Illness. Available at SSRN 3580524. 2020.
20. Lespine A, Alvinerie M, Sutra JF, Pors I, Chartier C. Influence of the route of administration on efficacy and tissue distribution of ivermectin in goat. Vet Parasitol. 2005;128(3-4):251-60.
21. Overbeck S, Rink L, Haase H. Modulating the immune response by oral zinc supplementation: a single approach for multiple diseases. Arch Immunol Ther Exp (Warsz). 2008;56(1):15-30.
22. Lanke K, Krenn BM, Melchers WJ, Seipelt J, van Kuppeveld FJ. PDTC inhibits picornavirus polyprotein processing and RNA replication by transporting zinc ions into cells. J Gen Virol. 2007;88(Pt 4):1206-17.
23. Liu CY, Kielian M. Identification of a specific region in the e1 fusion protein involved in zinc inhibition of semliki forest virus fusion. J Virol. 2012;86(7):3588-94.
24. Shishkov S, Varadinova T, Bontchev P, Nachev C, Michailova E. Complexes of zinc with picolinic and aspartic acids inactivate free varicella-zoster virions. Met Based Drugs. 1996;3(1):11-4.
25. Gaudernak E, Seipelt J, Triendl A, Grassauer A, Kuechler E. Antiviral effects of pyrrolidine dithiocarbamate on human rhinoviruses. J Virol. 2002;76(12):6004-15.
26. Denison MR, Zoltick PW, Hughes SA, Giangreco B, Olson AL, Perlman S, et al. Intracellular processing of the N-terminal ORF 1a proteins of the coronavirus MHV-A59 requires multiple proteolytic events. Virology. 1992;189(1):274-84.
27. Ferrari E, Wright-Minogue J, Fang JW, Baroudy BM, Lau JY, Hong Z. Characterization of soluble hepatitis C virus RNA-dependent RNA polymerase expressed in Escherichia coli. J Virol. 1999;73(2):1649-54.
28. Hung M, Gibbs CS, Tsiang M. Biochemical characterization of rhinovirus RNA-dependent RNA polymerase. Antiviral Res. 2002;56(2):99-114.
29. Te Velthuis AJ, van den Worm SH, Sims AC, Baric RS, Snijder EJ, van Hemert MJ. Zn2+ inhibits coronavirus and arterivirus RNA polymerase activity in vitro and zinc ionophores block the replication of these viruses in cell culture. PLoS pathogens. 2010;6(11).
30. Perlman S, Netland J. Coronaviruses post-SARS: update on replication and pathogenesis. Nature reviews Microbiology. 2009;7(6):439-50.
31. Lu R, Zhao X, Li J, Niu P, Yang B, Wu H, et al. Genomic characterisation and epidemiology of 2019 novel coronavirus: implications for virus origins and receptor binding. Lancet (London, England). 2020;395(10224):565-74.
32. Latapiat V, Rodríguez FE, Godoy F, Montenegro FA, Barrera NP, Huidobro-Toro JP. P2X4 receptor in silico and electrophysiological approaches reveal insights of ivermectin and zinc allosteric modulation. Frontiers in pharmacology. 2017;8:918.
33. Arigony AL, de Oliveira IM, Machado M, Bordin DL, Bergter L, Pra D, et al. The influence of micronutrients in cell culture: a reflection on viability and genomic stability. Biomed Res Int. 2013;2013:597282.
34. Assis D, Lim J. New pharmacotherapy for hepatitis C. Clinical Pharmacology & Therapeutics. 2012;92(3):294-305.
35. Harika R, Faber M, Samuel F, Kimiywe J, Mulugeta A, Eilander A. Micronutrient Status and Dietary Intake of Iron, Vitamin A, Iodine, Folate and Zinc in Women of

Reproductive Age and Pregnant Women in Ethiopia, Kenya, Nigeria and South Africa: A Systematic Review of Data from 2005 to 2015. Nutrients. 2017;9(10).
36. Shivakoti R, Christian P, Yang W-T, Gupte N, Mwelase N, Kanyama C, et al. Prevalence and risk factors of micronutrient deficiencies pre-and post-antiretroviral therapy (ART) among a diverse multicountry cohort of HIV-infected adults. Clinical nutrition. 2016;35(1):183-9.
37. Mamo JC, Naissides M. Could iodine be effective in the treatment of human immunodeficiency virus and AIDS-associated opportunistic infections? Int J Infect Dis. 2005;9(5):292-3.
38. Sriwilaijaroen N, Wilairat P, Hiramatsu H, Takahashi T, Suzuki T, Ito M, et al. Mechanisms of the action of povidone-iodine against human and avian influenza A viruses: its effects on hemagglutination and sialidase activities. Virology journal. 2009;6(1):124.
39. Levine M, Padayatty SJ, Espey MG. Vitamin C: a concentration-function approach yields pharmacology and therapeutic discoveries. Adv Nutr. 2011;2(2):78-88.
40. Lima AF, Lessa FC, Mancini MN, Hebling J, Costa CA, Marchi GM. Transdentinal protective role of sodium ascorbate against the cytopathic effects of H2O2 released from bleaching agents. Oral Surg Oral Med Oral Pathol Oral Radiol Endod. 2010;109(4):e70-6.
41. Wilson R, Mc Killop J, Thomson J. The effect of pre-operative potassium iodide therapy on antibody production. European Journal of Endocrinology. 1990;123(5):531-4.
42. Pan F, Ye T, Sun P, Gui S, Liang B, Li L, et al. Time course of lung changes on chest CT during recovery from 2019 novel coronavirus (COVID-19) pneumonia. Radiology. 2020:200370.
43. Franquet T. Imaging of pulmonary viral pneumonia. Radiology. 2011;260(1):18-39.
44. Koo HJ, Lim S, Choe J, Choi S-H, Sung H, Do K-H. Radiographic and CT features of viral pneumonia. Radiographics. 2018;38(3):719-39.
45. Cascella M, Rajnik M, Cuomo A, Dulebohn SC, Di Napoli R. Features, evaluation and treatment coronavirus (COVID-19). StatPearls [Internet]: StatPearls Publishing; 2020.
